# Virtual agents as a scalable tool for diverse, robust gesture recognition

**DOI:** 10.3758/s13428-025-02914-w

**Published:** 2026-01-16

**Authors:** Lisa Loy, James P. Trujillo, Floris Roelofsen

**Affiliations:** University of Amsterdam, Science Park 900, Amsterdam, 1098 XG UK

**Keywords:** Gesture recognition, Synthetic data, Virtual agents, Avatars

## Abstract

Gesture recognition technology is a popular area of research, offering applications in many fields, including behaviour research, human–computer interaction (HCI), medical research, and surveillance culture, among others. However, the large quantity of data needed to train a recognition algorithm is not always available, and differences between the training set and one’s own research data in factors such as recording conditions and participant characteristics may hinder transferability. To address these issues, we propose training and testing recognition algorithms on virtual agents, a tool that has not yet been used for this purpose in multimodal communication research. We provide an example use case with step-by-step instructions, using mocap data to animate a virtual agent and create customised lighting conditions, backgrounds, and camera angles, creating a virtual agent-only dataset to train and test a gesture recognition algorithm. This approach also allows us to assess the impact of particular features, such as background and lighting. Our best-performing model in optimal background and lighting conditions achieved accuracy of 85.9%. When introducing background clutter and reduced lighting, the accuracy dropped to 71.6%. When testing the virtual agent-trained model on images of humans, the accuracy of target handshape classification ranged from 72% to 95%. The results suggest that training an algorithm on artificial data (1) is a resourceful, convenient, and effective way to customise algorithms, (2) potentially addresses issues of data sparsity, and (3) can be used to assess the impact of many contextual and environmental factors that would not be feasible to systematically assess using human data

**Supplementary Information:**

The online version contains supplementary material available at 10.3758/s13428-025-02914-w.

## Introduction

Gesture recognition technology is a popular area of research, offering many application fields, including behaviour research, human–computer interaction (HCI) or human–robot interaction (HRI), medical research, surveillance culture, and many more (Hwang et al., 2006). The potential of gesture recognition technology as a tool should not be underestimated, especially for research on multimodal communication involving gestures and/or sign language. There has been a strong rise in multimodal communication research in recent years (Sparaci & Volterra, 2017). According to Kelmaganbetova et al. (2023), communication based on visual signals—such as gestures and facial expressions—is the most extensive and reliable form of communication, as it is not as planned and controlled as speech. Just like speaking, gesturing fulfils vital social actions (Kendon, 2017; Mondada, 2016) and is found across ages and cultures; even people born blind gesture, despite not ever having seen another person using gestures (Goldin-Meadow, 2000). Because most gestures are produced unintentionally, gestures can reveal valuable information about underlying subconscious mechanisms and are consequently essential in both linguistic and cognitive behaviour research. Investigating their influence on the way humans communicate is therefore a compelling endeavour.

Because there is no unified typology for gestures used in multimodal research (Kelmaganbetova et al., 2023), manual annotation of gestures is not only time-consuming, but it also leaves room for imprecision and disputes among coders due to ambiguities and subjective judgements. In order to mitigate such imprecision and reduce the time spent on manual annotation, Pouw et al. (2020) suggest employing motion tracking technology to automate the annotation and coding process while simultaneously obtaining detailed information about the spatio-temporal relations between speech and its accompanying gestures. Given that different researchers will have different research questions and data recording conditions, researchers should not only use existing algorithms—such as motion tracking or gesture recognition algorithms—to their advantage, but create their own specialised algorithms, tailored to their research interests and goals. For instance, if a researcher aims at investigating how a certain gesture is used to direct attention to a specific part of the accompanying information, she should not simply use existing gesture recognition or pose estimation technology but rather train a new algorithm on data involving the exact gesture under investigation to receive even more accurate and detailed results.

The starting point of gesture recognition is the collection of data to be used, whether for training an algorithm or for applying a trained model in order to automatically capture gestures. Typically, researchers want to apply gesture recognition to videos in order to leverage the recognition in real time, or to apply the recognition algorithm to existing videos. Pose estimation is typically the first step in processing the data, and involves the estimation of the position of multiple key points (e.g., body joints), which can then be used for gesture recognition. Pose estimation approaches can be divided into two categories and a combination thereof: video-based approaches and device-based approaches (e.g., Pouw et al., 2020; Trujillo, 2024). Video-based approaches offer researchers the advantage of being able to use it on preexisting videos and corpora; hence no new data need to be collected (Nyatsanga et al., 2023). This opens endless possibilities for employing gesture recognition technology even on corpora which were not originally intended for multimodal research. Furthermore, employing video-based tracking is an attractive option for researchers, as it is inexpensive, and many open-source programs are readily available (Pouw et al., 2020). Device-based approaches, on the other hand, are not readily available to all researchers, often requiring expensive equipment. However, they offer a higher spatio-temporal resolution than video-based tracking (Pouw et al., 2020). Furthermore, device-based recordings have smaller information loss than video-based systems, as most devices capture poses and movement in 3D rather than 2D (but see recent advances in 3D tracking from single-view 2D video, e.g., Pavlakos et al., 2024; Rong et al., 2021), which further reduces the effect of (self-)occlusion on recognition accuracy and provides the researcher with 3D ground truth data.

Reliable gesture recognition depends not only on the type of data used (e.g., video- or device-based, as described above), but also on the architecture used for classification and the number and granularity of gesture categories being classified. For example, previous studies have achieved a mean accuracy score of 90.8% on six hand gestures collected with Microsoft Kinect (Ding & Su, 2023), 93.1% accuracy using a pretrained convolutional neural network (CNN) and transfer learning across 11 hand gestures (Hussain et al., 2017), and 97.3% for eight gestures using a Perception Neuron motion capture suit (Shanthakumar et al., 2020). While accuracy can be quite high, a recent survey of gesture recognition work from 2018 to 2023 reported that accuracy ranges from 52% to 98%, with an average of 79% accuracy (Hashi et al., 2024). The authors also report that large datasets are required to achieve high accuracy.

As with every data-driven approach, both video-based and device-based approaches to gesture recognition algorithms are fundamentally limited by the data they are trained on (Nyatsanga et al., 2023). It is often difficult to find enough data involving the exact phenomenon a researcher plans to investigate which is sufficiently diverse in terms of participant demographics to efficiently train an algorithm, even with the abundance of data found online. Especially in times of big data, the quality of (training) datasets has an enormous impact on the performance of machine learning (ML) models (Gong et al., 2023). For instance, the training data can lead to unwanted bias in the resulting algorithm if the training data are skewed in any way or lack diversity (e.g., De Cremer & Kasparov, 2022; Prabhumoye et al., 2019; Mikalef et al., 2022). It is therefore of utmost importance to create a balanced and inclusive dataset to prevent such biases from forming. Although the use of transfer learning—i.e., using a model which is pretrained on a larger, related dataset and then retrained on a smaller, more specialised dataset—reduces the dataset size required to effectively train a new ML model considerably, it is still crucial that the dataset, albeit smaller, is inclusive and diverse. This may be related to factors such as recording conditions, which may be very different from the typical front or top view seen in many gesture recognition databases when researchers are collecting naturalistic data in the field, or even unconstrained conversation.

### Leveraging virtual agents to tackle the problems of data sparsity and data diversity

As a solution to the data sparsity problem, we suggest harnessing the advantages of avatars, or virtual agents, to create synthetic data. As already mentioned, collecting enough data using human subjects, particularly when trying to account for or even quantify the impact of contextual factors (e.g., lighting, background, camera angle), is not always possible. However, current advances in technology allow researchers to create human-like virtual agents, which can be used to imitate human participants and create synthetic data. This approach allows researchers to mitigate not only the overall absence of available data, but also the lack of diversity in the data that are available. There are endless possibilities for customising said virtual agents, their behaviour, their surroundings, and many more aspects. This additionally allows one to quantify or assess the impact of particular features, such as lighting or camera angle, without recording new datasets for each variation.

The use of synthetic data has gained considerable traction recently in the fields of computer vision (CV) and machine learning (ML) more generally (see, e.g., Hittmeir et al., 2019; Nikolenko, 2021; Borkman et al., 2021; Tripathi et al., 2019). This is not surprising, as datasets built on artificial data offer vast possibilities to researchers in many domains, including multimodal communication research. However, research on multimodal communication to date has not yet applied this approach. To change this, the current study leverages the advantages of using virtual agents to train a recognition algorithm to facilitate the creation of customised recognition algorithms for research purposes, creating a sizable dataset consisting of only virtual agent recordings to train a gesture recognition algorithm. The research presented here will address the question of whether a dataset consisting only of virtual agents can be successfully used to train a gesture recognition algorithm. Furthermore, it investigates whether common pitfalls of gesture recognition algorithms observed in the literature—such as poorly lit frames, cluttered backgrounds, and problematic angles (e.g., Pouw et al., 2020; Nyatsanga et al., 2023; Gong et al., 2023)—persist if a gesture recognition algorithm is trained on a synthetic dataset. We first outline how virtual agents are used in the current literature before then suggesting how they could and should be used in future research. We furthermore provide an overview of some of the most influential aspects on the accuracy of gesture recognition algorithms and how virtual agents help researchers either reduce the negative effects of these aspects or adjust these aspects according to their research interests. Finally, we illustrate the validity of this approach by presenting a use case, building a prototype for a gestural emergency call, and discussing the suitability of a virtual agent-only dataset for the creation of a gesture recognition algorithm intended for use on human beings.

### Leveraging virtual agents

Virtual agents are employed for a variety of purposes, which are also reflected in research, starting from purely entertaining purposes, such as building a pipeline which automatically creates a virtual agent similar to emojis from an image of a user (Wolf et al., 2017), and virtual agents used for HCI (e.g., Ahn et al., 2004; Hung et al., 2022), all the way to using virtual agents as educational agents (e.g., Agrawal et al., 2011; Haginoya et al., 2023) or as a means to translate from text to sign language (e.g., Roelofsen et al., 2021; Van Gemert et al., 2022; Nolte et al., 2023; De Martino et al., 2023). In a survey on the use of virtual agents, Atasoy et al. (2023) found that 28 out of the 79 virtual agent papers they identified used their virtual agents for scientific studies, and 47 used their virtual agents for mobile and web applications for sign language interaction and translation. A notable example of virtual agents used as a middle product (i.e., not the input to an algorithm, but also not an end product in itself) is the generation of virtual agents as experimental stimuli (Gratch, 2023; Pan & Hamilton, 2018; Peeters, 2019). These may be non-interactive, rendered videos that allow for more control and experimental manipulation than natural videos (Nölle et al., 2021; Nota, Trujillo, Holler et al., 2023a, Nota, Trujillo, Jacobs et al., 2023b; Snoek et al., 2023; Wu et al., 2024) or interactive agents used to study social interaction (Aburumman et al., 2022; Hale & Hamilton, 2016; Hömke et al., 2018).

While many studies create virtual agents as a ‘final product’ (e.g., as a sign language interpreter) or as stimulus material in experiments, only a few studies have harnessed the advantages of virtual agents as input for an algorithm. Zhang et al. (2020), one of the few studies including actual whole-body virtual agents to train a recognition algorithm at the time of writing, used a combination of three datasets to train their hand pose estimation algorithm:

1. A human in-the-wild dataset with varying backgrounds and lighting conditions,
2. A human in-house-collected dataset with varying angles but consistent backgrounds, and
3. An artificially created synthetic dataset with virtual agent-like hands in front of varying backgrounds.

**Fig. 1:**
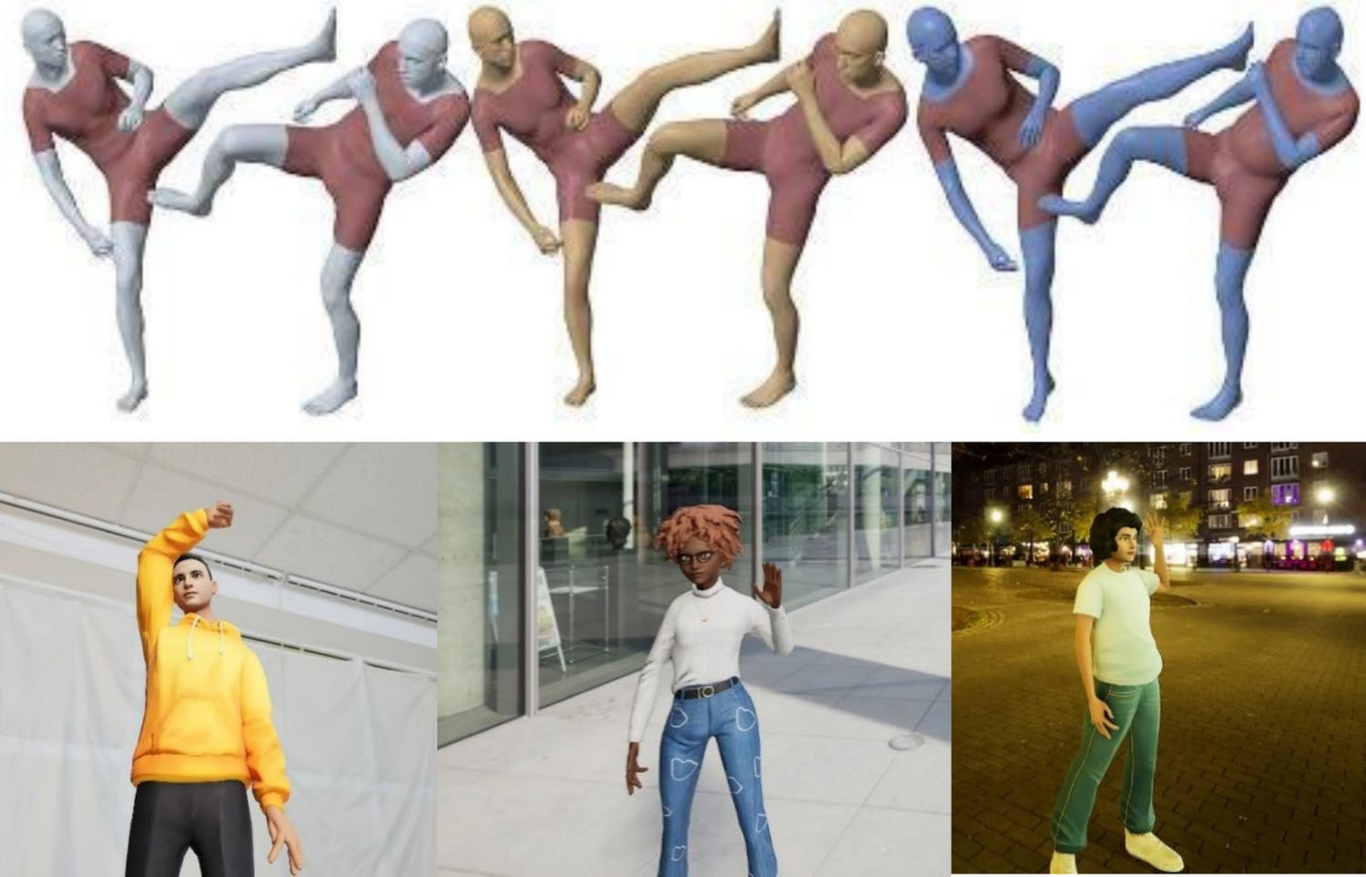
Comparison of Xu et al.'s (2020, p. 6191) characters (top three pairs) and the virtual agents created in the current project (bottom three)

To create these artificial hands, the authors use Xu et al.’s (2020) generic human shapes model pipeline. The pipeline, trained on a large dataset of 60,000 high-resolution photo-realistic dynamic complete 3D body scans, creates human-like characters, similar to avatars. Xu et al.’s model is able to reproduce various facial expressions, body shapes, and hand shapes on their gender-neutral characters. However, the virtual agents produced by Xu et al. (2020) and used by Zhang et al. (2020) do not directly resemble humans in real video recordings. Although the pipeline reconstructs 2D images of humans to 3D representations on a virtual character, the characters themselves do not have the typical human skin tones, do not have eyes, do not have hair, and do not have customisable clothing (see Fig. 1 for a comparison with the virtual agents used in the present study). This makes it challenging to use such virtual agents to address challenges faced by behavioural researchers with more naturalistic data, such as different lighting, skin tone, or background.

Similar to the approach by Zhang et al. (2020), Ranum et al. (2024a, b) constructed a dataset containing videos of signs in Sign Language of the Netherlands (Nederlandse Gebarentaal, NGT), combining video recordings of three human native NGT signers and one synthetic avatar signer, to train a multi-view sign recognition algorithm. They only used one virtual character and one neutral background, rendered from three different angles. Thus, while the studies by Zhang et al. (2020) and Ranum et al. (2024a, b) both employ synthetic data to supplement human data, they do not yet leverage the full potential of virtual agents. Creating a completely artificial dataset, customising both the virtual agents themselves and their environment in various ways, offers multiple advantages.

### Control through customisability

As mentioned earlier, especially when it comes to niche phenomena or low-resource languages, the data available are scarce. Therefore, researchers cannot always choose what the data they want to collect look like or under what circumstances the data are collected. This makes it difficult to control for, or sometimes even anticipate, the influence of certain aspects on the outcome of the studies. Existing gesture recognition research has found that there are multiple aspects potentially affecting the accuracy of a recognition algorithm. Using a synthetically created dataset based on virtual agents allows researchers to better understand or control for common issues recognition technology still faces. This approach not only helps researchers avoid common pitfalls, potentially leading to better recognition accuracy, but also challenges and provokes said pitfalls. In a best-case scenario, a gesture recognition algorithm performs consistently well across gestures performed in different environments—including difficult lighting conditions and cluttered/low-contrast backgrounds, among others—and performed with varying speed, amplitude, visibility, etc. However, this is unfortunately not yet the case. Challenging gesture recognition algorithms by intentionally introducing problematic data enables researchers to identify weaknesses in the pipeline, which can then be further revised and advanced, leading to the improvement of the overall gesture recognition algorithm over time. Alternatively, the researcher may choose which aspects of their data collection protocol to adjust in order to most efficiently improve data quality.

#### Participant diversity

As mentioned previously, a recurring theme in the literature on ML and big data is the lack of diversity in the datasets used to train algorithms (Bender et al., 2021). According to Hovy and Spruit (2016), most biases in ML algorithms develop as the datasets they are trained on are curated using data coming from a mainly educated, industrialised, rich, western, white male participant database. Due to the nature of ML algorithms, the resulting models and applications are most efficient on data similar to those they have been trained on. Therefore, the applications unintentionally discriminate against users who do not conform to what the algorithms have been trained on. Even though Sánchez-Brizuela et al. (2023) claim to have created an algorithm robust against variation in skin tones using dynamically calculated ranges to separate hand borders and similarly coloured backgrounds, their algorithm only works efficiently in what they call ‘nonextreme’ cases. In their discussion, the authors admit the algorithm frequently malfunctions in sub-optimal conditions, most likely due to sensor saturation. These limitations found in the literature emphasise the importance of creating a varied training dataset. By creating a dataset employing virtual agents, researchers can adjust the skin tones of the virtual agents to a range of different shades to account for as many skin tones as possible during training. Using this feature, researchers can not only ensure their own model is trained on a diverse dataset, therefore actively preventing biases from forming, but also test existing models to investigate whether they suffer from such biases.

#### Settings and surroundings

Another aspect strongly affecting recognition accuracy is the environment the data are captured in. The background composition in particular is known to cause problems as ML algorithms struggle to correctly recognise hand shapes if the contrast between the hand and the background is not sufficient (e.g., Nyatsanga et al., 2023; Sanchez-Brizuela et al., 2023). Video-based tracking systems are especially vulnerable to cluttered backgrounds and may not be able to pick up on small movements of the hands/fingers if the contrast is too low (Pouw et al., 2020). Just as with humans, the recognition algorithms need to be able to differentiate between a virtual agent and its background to be able to recognise the gesture a virtual agent is performing. However, the researcher can purposefully include as many cluttered backgrounds as possible in the training data to try and accustom the algorithm to such conditions to mitigate these recognition issues in future applications. Additionally, the researcher can artificially create scenarios which are difficult to encounter in real life. Using techniques such as employing high dynamic range images (HDRIs) enables the researcher to position her virtual agents anywhere in the world. This may be in environments where actual real-life data collection is not feasible, or where data collection is expected to take place, allowing the researcher to assess feasibility in terms of data quality.

#### Lighting conditions

Similar to the environment, the lighting conditions in which the images are captured have a substantial impact on the recognition accuracy of an algorithm. In particular, methods based on colour differences between hands and background, such as using a pixel difference threshold, are easily affected by fluctuating lighting conditions (Sánchez-Brizuela et al., 2023). Varying lighting conditions have been shown to lead to erroneous segmentation between hands and background for both video-based and device-based recognition algorithms (e.g., Agrawal et al., 2011; Liu & Peng, 2009). As most rendering software offers the option to control the artificial light in the virtual agent scene, researchers can, again, use this ability to customise the lighting conditions to avoid any issues with imbalanced light. Alternatively, the researcher can purposefully adjust any light sources to imitate different times of day or different weather conditions to try and accustom the algorithm to such conditions. Training the algorithm in well-lit and balanced conditions does not represent reality, as most data collected in the wild are captured in varied lighting conditions and during different times of the day. Therefore, including a variety of lighting conditions in one’s dataset reflects reality more accurately. Additionally, adjusting reflections and shadows to resemble naturalistic conditions has been shown to help virtual agents appear more realistic (Atasoy et al., 2023).

#### Camera angles

In addition to using a naturalistic setting, including lighting and shading, it is more realistic to show a person not just from the front, but also from different angles. This is because when collecting data ‘in the wild’, human participants would most likely not be directly facing the camera at all times unless explicitly instructed to do so. Therefore, virtual agents should aim at reproducing uninhibited human behaviour, meaning one should also capture virtual agents from different angles to capture as many facets of the movements as possible. Furthermore, despite usually reaching high accuracy of around 90%, vision-based methods are often affected by environmental factors, such as the number and capturing range of cameras (Shanthakumar et al., 2020). Including frames of a gesture captured from different angles and distances in the training data therefore improves the robustness of the resulting algorithm. Most rendering programs offer the researcher the option to determine the distance of the virtual agent from the camera as well as the angle with which the camera points at the virtual agent. Researchers can leverage the ability to adjust these camera properties to imitate various recording settings. These settings include for example, as is often the case nowadays, bystanders filming from the sidelines, or using CCTV cameras to collect the data. An additional advantage of being able to adjust the camera angle is mitigating self-occlusion. According to Kurakin et al. (2012), hand gestures are much more difficult to recognise than whole-body gestures, as hand gestures are more subtle with smaller movements, often suffering from serious occlusions between individual fingers. Supporting this claim, Forte et al. (2023) found that self-contact of the fingers leading to self-occlusions as well as motion blur are among the most striking challenges during automated sign recognition. Therefore, being able to adjust the angle and proximity of the virtual agent gives the researchers the freedom of deciding how to mitigate these issues.

#### Artificial variance

Another factor in resolving the data sparsity problem is the ability to artificially create variance in the data. Although most virtual agents are created by an automated process using a pipeline, directly projecting the animations created through keyframe synthesis or mocap recordings onto a basic virtual agent, they can still be modified manually. Because the recording of mocap data is not only time-consuming but also expensive, the mocap data of one person are commonly projected onto multiple virtual agents. This prevalent approach was criticised by Narang et al. (2017), claiming that this results in the virtual agent and its movements only being representative of one person rather than a variety of people. While this might be true in its essence, this issue can be mitigated to some extent by manually creating artificial variance. For instance, researchers could slightly adjust the positioning of individual fingers to create a new variation of a gesture. Furthermore, the gesture height can easily be modified by adjusting the position of the virtual agent’s arm using forward kinematics. By combining different slight adjustments to the original recording, the researcher can artificially create endless new combinations and varieties of an existing gesture. This variance in the data can moreover help with training a more robust algorithm. During the formation of their sign recognition algorithm, Moryossef et al. (2020) discovered that their pose estimation algorithm assigned both wrist key points high weights, with the right hand being weighted even higher. The authors argue that this might be due to most people being right-handed. By creating more left-handed virtual agents to train the algorithm on, this bias towards the right wrist/hand in general could be resolved to assign (near) equal weight to both the virtual agent’s left and right hands. The ability to customise the virtual agent and its animations to fit one’s research interests enables researchers to explore such possibilities.

## Use case: A gestural emergency call

In the preceding sections we have outlined current approaches and issues with training gesture recognition algorithms and suggested leveraging virtual agents to create datasets to train and test gesture recognition algorithms. In the next section, we illustrate the validity of this approach by presenting a use case, employing virtual agents to create a large-scale dataset to train a MediaPipe gesture recognition algorithm used to build a gestural emergency call prototype.

### The ‘Signal for Help’ gesture

**Fig. 2:**
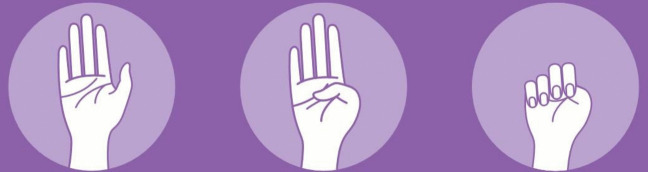
Signal for Help gesture (Canadian Women's Foundation, 2023)

On 8 November 2021, newspapers around the globe (e.g., Victor & Medina, 2021; Cardoen, 2021; Ritschel, 2021) reported on a kidnapped girl in the United States being found by the police after having used a hand gesture she had previously learned on the social media platform TikTok. The one-handed gesture (see Fig. 2) is known as the ‘Signal for Help’ gesture (henceforth SfHg), created in 2020 during the COVID-19 pandemic by the Canadian Women’s Foundation as a discrete sign for victims of domestic violence to use during video calls.

The gesture is formed by a sequence of three handshapes: an open palm with stretched fingers followed by only the thumb being tucked in and ending in the thumb being enclosed by all other fingers, resembling a fist. Despite, or even due to, the gesture being fairly simple yet effective, it has spread around the world and has gone viral on many social media platforms, likely helping countless victims.

Using gestures to call for help rather than doing so vocally can aid not only people with conditions such as mental and/or physical disabilities or other inhibiting factors (Kendon, 2017; Mondada, 2016), but also people in dangerous situations—for example, if a victim’s predator is present, and calling for help vocally is not safe. Even though today’s technology allows for innovations like an international emergency alert based on gestural input, such a system does not exist yet. The need for an inclusive, accessible, and discrete way to alert the authorities and call for help is undeniable. We therefore make use of the accessibility and simplicity of the SfHg to train a custom gesture recognition algorithm, on which such a visual-gestural emergency call (henceforth GEC) can be built.

## Method

### Study design

Using the surveillance culture now prevalent in most countries as an advantage, we could imagine building a GEC that is intended to be installed not only on personal devices, such as phones, laptops, and other mobile devices, but also in closed-circuit TV (CCTV) surveillance cameras, for example in elevators, public locations like shopfronts, and public transport. However, to create such an alarm, an enormous corpus of data representative of all the aforementioned areas of application is needed. To create this corpus, we apply the methodology we have discussed in the Introduction, and which we further detail in the following paragraphs, to compile a corpus based purely on artificial data using virtual agents. Using mocap technology, we recorded animation data of the SfHg performed in ‘naturalistic’ ways. Following the mocap recordings, humanlike virtual agents were created and animated using Unreal Engine (Epic Games, 2019) to perform either unrelated gestures (distractors) or the SfHg. The resulting videos of the virtual agents were adjusted and rendered to account for a variety of features, including the virtual agents’ characteristics (e.g., skin tone, gender), as well as the environment (e.g., camera angle, background). Thereafter, MediaPipe (Lugaresi et al., 2019), an open-source pose estimation software, was employed to train a deep learning algorithm to automatically scan the synthetic videos for the gestures present. Once established, the algorithm was trained and validated before being tested and evaluated using common evaluation metrics. If the trained model performs well—suggested by high scores for accuracy, precision, and recall—this provides evidence that virtual agents can indeed imitate human data well enough, therefore answering the question of whether it is possible to successfully train a gesture recognition algorithm on a dataset consisting only of virtual agents. However, low scores for the aforementioned evaluation metrics would indicate that either (a) despite the thorough and intricate design of the virtual agents, the algorithm did not learn the gestures correctly if at all, or (b) there are issues with the recognition algorithm which are not directly caused by the virtual agents but by the artificially created environmental factors, such as lighting, angle, and background. By contrasting model performance across datasets with different environment features, we can learn whether particular environmental factors led to poorer performance.

Assuming the model and task work well, a prototype of the GEC is programmed to illustrate prospective next steps and give an example of how to use recognition algorithms trained on virtual agents and apply them on humans. To do so, the gesture recognition task will be extended to scan the recognition output and automatically alert the authorities if the SfHg is detected.

#### Motion capture recordings

We recorded the gestures and distractors using a mocap setup similar to that of Ranum et al. (2024a, b). Recording took place at the Visualisation Lab of the University of Amsterdam in the Netherlands. The mocap studio includes a Vicon rig composed of 10 Vicon Vero v2.2 optical motion capture cameras (Vicon Motion Systems Ltd, 2016) installed on the ceiling, and two cameras located on the floor directly in front of the person performing the gesture (henceforth gesturer). The gesturer was fitted with a full-body suit using the standard 53-marker set Vicon FrontWaist template. Additionally, we used the StrechSense (model: Pro Fidelity) gloves (StretchSense, 2022). The gloves are equipped with Hand Engine Pro and powered by a Dell Universal Dock (UD22) and one USB dongle for each glove. See Andersen et al. (2025) for a more detailed overview of the lab set-up, including hardware and technical details.

Calibration of the Vicon setup followed the built-in Vicon calibration protocol to ensure all cameras were activated. We rendered the motion data directly onto an initial virtual agent in Unreal Engine using Shogun Live and Shogun Post (v.1.11). The calibration process built in the Hand Engine Pro software for the StrechSense gloves involved two steps: Express Calibration and Advanced Calibration. During the Express Calibration process, the gesturer is asked to imitate four key poses. The Advanced Calibration allows the researcher to upload a custom pose library. For the current study, a library containing the most common handshapes found in NGT was used. The gesturer repeats movements using different handshapes as shown on a screen. Once the handshape is imitated, the gesturer is asked to rotate the wrist for the gloves to capture the whole motion. The complete process, including Express and Advanced Calibration, was performed in blend pose mode. During recording, version 3.0.6 of the Hand Engine Pro software (StretchSense, 2022) was used.

For the mocap part of this study, one participant was recorded. In total, we captured six distractor-only recordings, 20 recordings of the SfHg only (10 performed with the left hand, 10 with the right hand), and 10 recordings of a distractor followed by the SfHg on alternating hands, resulting in 36 gestures being recorded overall. The gestures are based on instances of the gesture being used by individuals on social media and are created by combining a hand orientation—right side up, upside down, sideways—with the height where the gesture is being performed—hip height, waist height, shoulder hight, next to head, overhead—resulting in 10 possible combinations (see Table 1). The distractors were chosen randomly and consist of common everyday gestures (e.g., shoulder shrugging, finger counting). The recordings were then retargeted directly into Unreal Engine animation assets.

**Table 1:** Combinations of hand orientation (*x*-axis) and gesture height (*y*-axis) used during mocap recordings

Possible combinations	Right side up	Sideways	Upside down
Hip height	X	✓	✓
Waist height	X	✓	✓
Shoulder height	✓	✓	X
Next to head	✓	✓	X
Overhead	✓	✓	X

#### Virtual agent creation

**Fig. 3:**
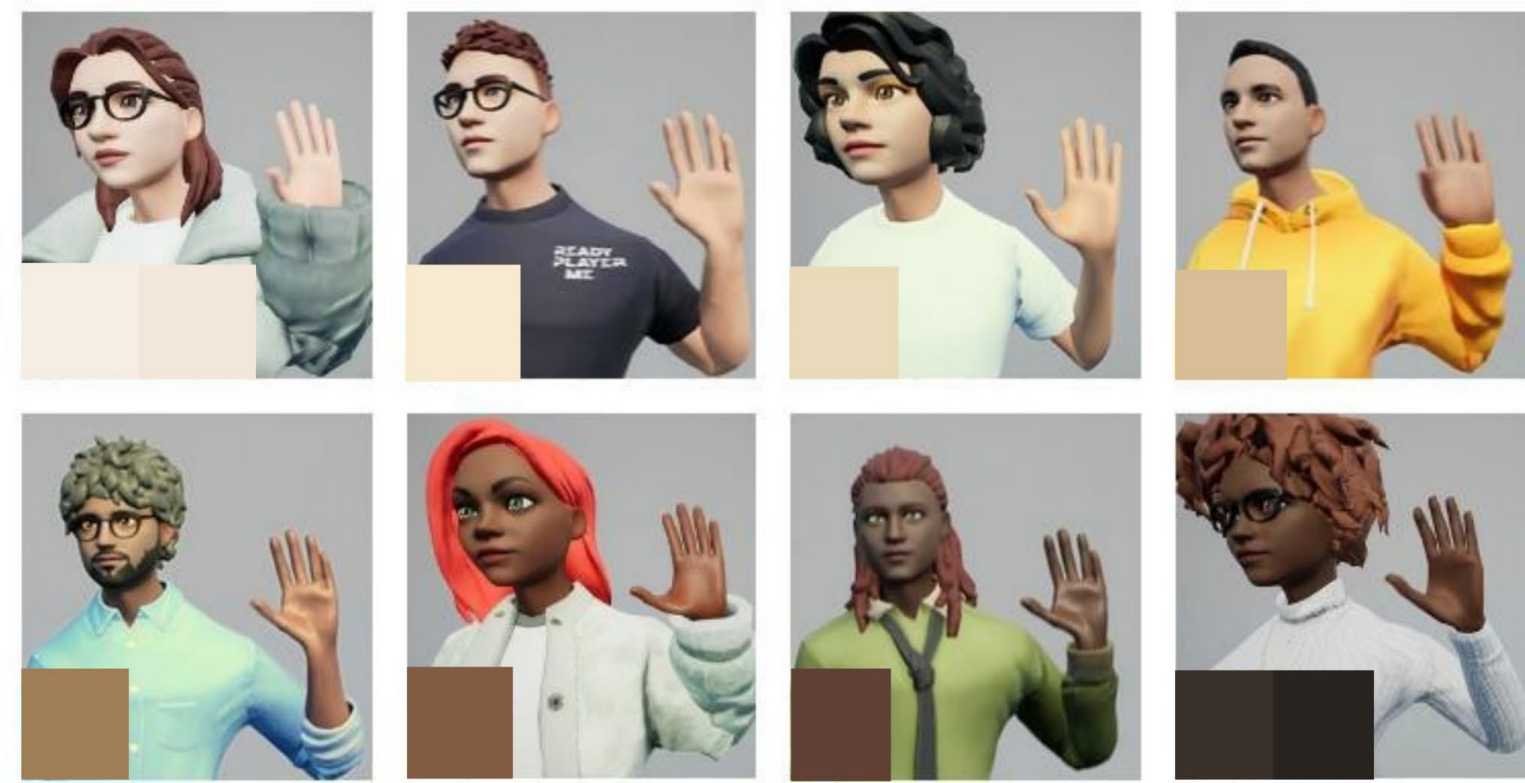
Virtual agents used in the current study. The bottom left corner on each of the eight images shows the hue/s on the Monk Skin Tone Scale the virtual agents are representing

After recording the gestures, we created eight different characters, four male and four female, based on the 10 hues on the Monk Skin Tone Scale, which was created to improve inclusivity and diversity in the field of ML (Monk, 2023, n.d.). All virtual agents were created using the Ready Player Me Avatar Creator (Ready Player Me, n.d.). The Avatar Creator allows the user to customise several aspects of the virtual agent, starting with the sex of the virtual agent, the skin tone, the eye/nose/mouth shape, eye/hair colour, and figure, all the way to the clothing the virtual agent is wearing. We tried to match the skin tones of the virtual agents as closely as possibly to the 10 hues in the Monk Skin Tone Scale to incorporate as much diversity as possible. However, as the software still only has a limited selection of attributes for the characters at the time of writing, we combined the first two skin tones of the scale and the last two tones of the scale as, according to Google’s MediaPipe documentation (2023a), there are fewer people perceived to be on either end of the scale than somewhere in between. Of course, these characters do not fully cover the diversity found in the human population. However, we believe that using eight different shades for the skin colours still creates more diversity than found in most other commonly used datasets. The eight resulting virtual agents can be seen in Fig. 3. Any of the other aspects of the virtual agents besides skin tone were chosen randomly, as it was determined that they would not affect the recognition accuracy in any way. Following the customisation process, the user can download the finished virtual agent as a.glb file, which can then be imported directly to Unreal Engine.

Animating the female virtual agents required one additional step of retargeting the original recordings onto the dimensions of a female character’s skeleton. Next, we fixed the ‘enclosed thumb’ handshape. Because the StrechSense gloves did not fit the gesturer’s hands perfectly, the virtual agents’ hands did not fully close for the enclosed thumb handshape. This was fixed manually using the forward kinematics control rig by adding an additional layer over the original to bake (blend) the corrected finger positioning to the original animation. This was done for all 30 animation assets containing the SfHg. Furthermore, also due to the glove fit issue, the virtual agents’ fingers were spread too far apart in the open palm handshape. As performing the gesture with spread fingers is not necessarily wrong and could be performed that way by human users, only half of the animation assets containing the SfHg (i.e., 15 animation assets) were corrected manually using the forward kinematics control rig. This created artificial variations of the gesture, resulting in one half of the gestures being performed with spread fingers and the other half being performed with the fingers closer together.

#### Dataset generation

After animating the virtual agents and fixing any issues and inaccuracies, we adjusted the environment. Unreal Engine enables the user to use high dynamic range images (HDRIs) as the backdrop for the ‘scene’ the virtual agent is placed in. These images create a realistic environment and can be adjusted to the researcher’s needs. We used the online repository Poly Haven (Poly Haven, n.d.) to download four HDRI backdrops targeted towards the two potential applications of the GEC: for CCTV implementation and/or on personal devices. Two backdrops were chosen specifically to imitate CCTV contexts (City Light and City Dark), whereas the other two imitate the use of a personal device at home (Home Light and Home Dark). As seen by the backdrop names, each application area has one well-lit environment (Light) and one poorly lit environment (Dark). This was chosen to test how well the recognition algorithm performs in poor lighting conditions. Additionally, we used one ‘neutral’ grey backdrop as a control condition. Unreal Engine offers a range of different lighting objects to adjust the lighting and shadows of both the background and the character in the scene (see Unreal Engine Documentation, n.d., for more information). This way the researcher is able to create different lighting conditions. We set the source type of the HDRI Skylight to the Cubemap of the HDRI backdrop, which automatically creates naturalistic lighting according to the chosen HDRI map. Additionally, we adjusted the lights in both dark backdrops to better resemble the colour spectrum of the scene (adding orange for Home Dark and yellow for City Dark).

**Fig. 4:**
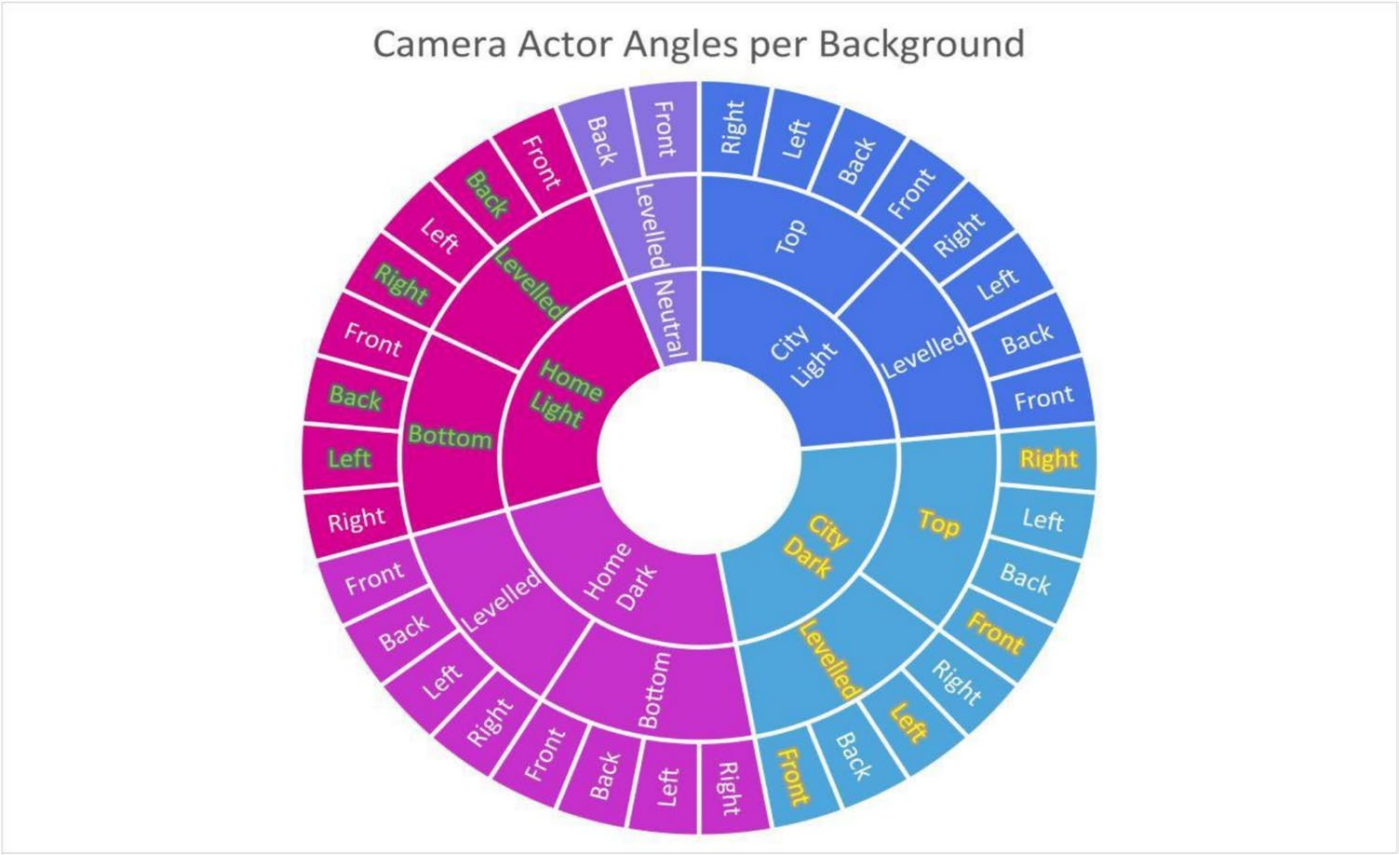
Illustration of all eight angles for the City and Home backdrops as well as the two angles for the neutral backdrop. Two examples of how the angles are combined: (1) gestures performed with the left hand and the palm facing the front in the City Dark background (highlighted in yellow) were captured from Levelled Front, Levelled Left, Top Front, and Top Right angles, and (2) gestures performed with the right hand and the palm facing backwards in the Home Light background (highlighted in green) were captured from Levelled Back, Levelled Right, Bottom Back, and Bottom Left

**Fig. 5:**
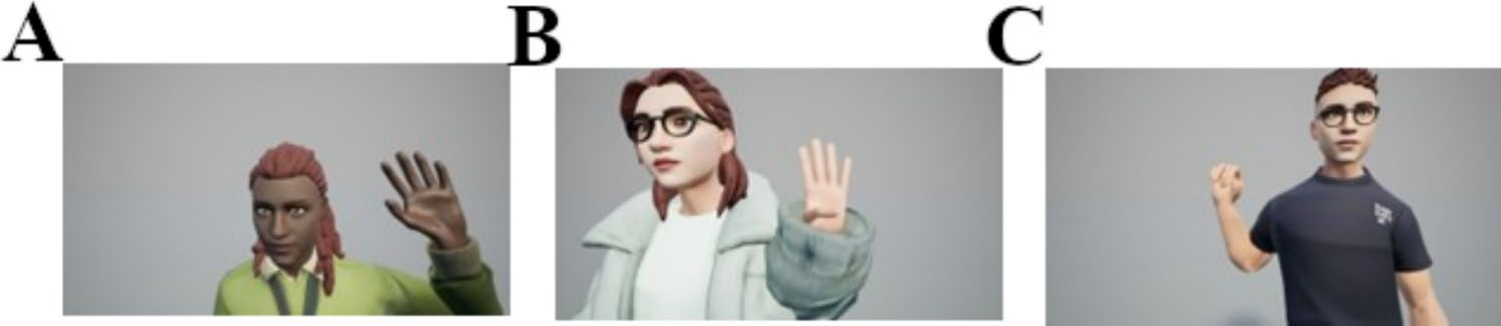
Examples of the three target handshapes: **A** open palm, **B** thumb tucked, and **C** enclosed thumb

Once the scene is set and ready for rendering, one must add a ‘Cine Camera Actor’ to the environment. This object works similarly to a real-life camera, and the user can adjust its location, zoom, and aperture, among other common camera features. The image seen through the camera actor is the scene to be rendered by Unreal Engine. We added eight different camera actors to the environment to account for eight distinct angles: Levelled Front, Levelled Back, Levelled Left, Levelled Right, Top Front, Top Back, Top Left, Top Right (all Tops for City backdrops only), Bottom Front, Bottom Back, Bottom Left, Bottom Right (all Bottoms for Home backdrops only). The neutral backdrop was only captured from Levelled Front and Levelled Back depending on the gesture. This is because the neutral backdrop was mainly used as the control condition; hence, aspects potentially affecting the recognition accuracy—such as bad lighting, cluttered backgrounds, and self-occlusion due to bad angles—are avoided with this backdrop, and only the straightforward levelled angles are used. For the remaining backdrops, each gesture was captured from four different angles. The levelled angles correspond to the palm orientation and the hand performing the gesture, whereas the additional angles from above or below correspond to the palm orientation and the opposite side of the hand performing the gesture (see Figs. 4 and 5 for illustration). This approach captured the gestures from all relevant angles while maintaining a concise study design. Capturing the gesture when not facing the camera was deemed irrelevant to the purposes of the current use case study, as all examples of the gesture being used in real life showed the person performing the gesture orienting the palm of their hand towards the camera/potential responder.

Following the process of setting up all cameras and corresponding angles, the scenes are imported to Unreal Engine’s Movie Render Queue. We rendered the scenes to frames with a rate of 15 frames per second. Supplementary Fig. 1 shows additional attributes of the render queue. In total, we rendered 144 scenes (36 gestures × 4 angles).

#### Frame selection

Once the rendering process was completed, the 10 most representative frames per handshape for gestures including the SfHg and the 15 most representative frames per handshape for the distractor gestures were chosen for the final dataset. By ‘most representative’ we mean frames where each handshape is clearly recognisable as such and there is no motion blurring. See Table 2 for an overview of the number of frames per handshape class in the full dataset.

**Table 2:** Distribution of frames per handshape class in the full dataset. The class ‘none’ contains the distractor gestures

Handshape Class	Number of Frames
**none**	6,210
**open palm**	42,350
**thumb tucked**	43,509
**enclosed thumb**	42,338

#### Gesture recognition algorithm training and optimisation

We used the Python implementation of MediaPipe’s convenient open-source Model Maker tool (MediaPipe, 2023a) to fine-tune MediaPipe’s Gesture Recognizer for the current dataset. Using a pretrained model and training it on a specialised dataset allows the researcher to train a high-performance model using both less data and less time. The model used is a fully connected neural network. While retraining, the last layers (classification layers) are rebuilt using the extracted features to fit the new categories (here open palm, tucked thumb, enclosed thumb, and none).

Retraining the Gesture Recognizer involves two main steps. As the Gesture Recognizer does not directly process input images, the code automatically runs MediaPipe Hands, a palm and finger tracking solution, on the dataset. This pipeline is a separate fully connected neural network with residual blocks, which detects the palm of a hand and extracts the *x*-, *y*-, and *z*-coordinates of 21 key points in the image space to create a landmark representation of the hand in the input image (Zhang et al., 2020). If the algorithm does not recognise a hand in the input image, it is discarded from the dataset. To regulate this dropout, the researcher can adjust the hand recognition confidence. For the purposes of the current study, we used the default hand recognition confidence of 0.7. The final output of this first step is a vector of length 128 representing the predicted hand landmark coordinates for each image in the dataset (MediaPipe, 2023b).

The resulting dataset containing, for each image in the original dataset, a vector representing the predicted hand landmark coordinates in that image is then split into three sub-datasets according to industry standards: 80% training data, 10% validation data, and 10% testing data (e.g., Li et al., 2019). These sub-datasets are then used to train the Gesture Recognizer. MediaPipe’s Model Maker enables its user to fine-tune a range of different hyperparameters, including the dropout rate, learning rate, batch size, and number of epochs (see MediaPipe, 2023b, for a complete list). A plethora of previous studies have shown that hyperparameter values significantly affect the performance of an ML model (e.g., Zhang et al., 2021; Weerts et al., 2020; Probst et al., 2019). However, the process of testing various value combinations and fine-tuning individual hyperparameters is costly and time-consuming. Given that the GEC implementation described here serves primarily as a proof of- concept for the virtual agents-as-gesture-recognition-input approach, we focused our analyses on four different hyperparameter configurations. The first configuration is the default settings of the MediaPipe Gesture Recognizer, while the second configuration was suggested as an alternative example on the MediaPipe Gesture Recognizer model customisation website (MediaPipe, 2023b). However, because the example provided by MediaPipe was trained on a dataset vastly different from ours, we suspected that different configurations may work better on the current dataset. As an additional test case, we added two configurations based on suggestions by ChatGPT (version 3.5) and Blackbox AI. Suggestions were given for the four hyperparameters (see Table 3) based on the size and file types of the data, as well as the target task for which the model would be trained. This approach provided not only an additional set of hyperparameters to test, but also allowed us to assess (albeit in a rather course-grained manner) the efficacy of using ChatGPT, an all-purpose chatbot based on generative AI (OpenAI, 2024) or Blackbox AI (version April 2024), a chatbot based on generative AI specifically trained to help with coding and development issues (Blackbox AI, 2024), for selecting (initial) hyperparameter configurations. A summary of the four hyperparameter configurations can be found in Table 3.

**Table 3:** Overview of configurations used to train the full model and the control model for the gesture recognition task

Configuration	Dropout rate	Learning rate	Batch size	Epochs
**MediaPipe default**	0.05	0.001	2	10
**MediaPipe example**	0.2	0.003	2	10
**ChatGPT**	0.4	0.001	64	50
**Blackbox AI**	0.3	0.005	128	20

In addition to the full model, we trained a second model on the well-lit neutral frames only, to function as a control model. The dataset used to train the second model consists of 7,911 frames (see Table 4), spread over the individual categories in a comparable ratio to the full dataset. The control model is trained to verify that any potential inaccuracies in the full model are due to general issues with the gesture recognition algorithm rather than any problems with the virtual agents. The second model was trained using the same four configurations as the full model.

**Table 4:** Distribution of frames per handshape class in the control dataset. The class ‘none’ contains the distractor gestures

Handshape Class	Number of Frames
**none**	360
**open palm**	2,154
**thumb tucked**	2,065
**enclosed thumb**	3,332

#### Programming GEC prototype

To demonstrate and test the system in practice, we implemented a simple proof of concept. Google’s manual for the creation of a gesture recognition task using Python (MediaPipe, 2023b) built on either MediaPipe’s pretrained model or the user’s own customised retrained model was used for the gesture recognition retraining. As the GEC is eventually intended to constantly scan camera images for the SfHg to call for help if needed, we used livestream as input for the gesture recognition task.

For the second part, we adjusted the script to check the recognition task’s output for the sequence in which the handshapes of the gesture occur. The code uses the output order together with the timestamps of the handshapes to verify that the sequence is indeed open palm, followed by thumb tucked, and ending in enclosed thumb. The code allows for empty recognition results in between two handshapes to account for the transition time between them. To calculate the transition time, we annotated a sample of the videos containing the SfHg found online in the annotation software ELAN (version 6.4 for Windows; ELAN, 2023). As the average person in the 10 sample videos found online uses around 0.86 s (ranging from 0.52 to 1.28 s) to transition from open palm to thumb tucked and on average 1.21 s (ranging from 0.93 to 1.68 s) transitioning from thumb tucked to enclosed thumb, we allowed a maximum of 2.0 s to still count as transition frames. Only if this exact sequence is recognised, the code will activate an alarm function, which is programmed to play the sound of an emergency vehicle siren. The sound file was downloaded from Mixkit, an online repository for publicly available sound effects (Mixkit, 2024). If the sequence is not recognised, recognised in the wrong order, or only partially recognised, the code keeps the alarm function deactivated. Furthermore, if the transition frames exceed 2.0 s, the gesture is considered to be cancelled, and the following handshape is counted as the first in a new sequence.

## Results

### Overall model performance

**Fig. 6:**
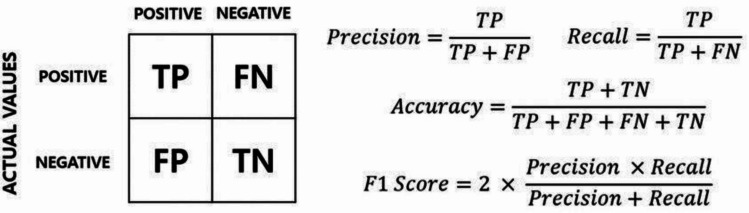
Formulas to calculate the evaluation metrics. TP = true positives, FN = false negatives, FP = false positives, TN = true negatives. Precision is calculated as the number of true positives divided by the total number of positive classifications of a given class. Recall is calculated as the number of true positives divided by the number of true positives and false negatives of a given class. F1 is calculated as precision × recall divided by precision plus recall, multiplied by 2. To illustrate how these formulas apply to the current study: when calculating the overall precision score, we first calculated the precision score for each handshape class and then the weighted average of these. Similarly for the overall recall score. For example, when calculating the precision for ‘open palm’, the label ‘open palm’ is the ‘positive’ label, and all other three labels are the ‘negative’ labels. When calculating the precision for ‘thumb tucked’, the label ‘open palm’ is now one of the three ‘negative’ labels, and the ‘positive’ label is ‘thumb tucked’. The same is the case for all four handshape classes in our data. All other metrics are calculated in the same manner

The original MediaPipe Model Maker template only includes test loss and categorical accuracy as their evaluation metrics. However, to gain a more holistic understanding of the strengths and flaws of the models, we evaluate all models on five metrics: loss, categorical accuracy, precision (P), recall (R), and F1-score (harmonic mean of precision and recall) (Wang & Li, 2019). As seen in Fig. 6, accuracy tells us, out of all the predictions the models make, how many are correct, whereas precision tells us, out of all the predictions of one class, how many actually belong to that class, and recall tells us, out of all the datapoints belonging to a certain class, how many the model found and how many were either not found or wrongly assigned to a different class.

### Predicted values

We first present the findings for the control model before moving on to the full model. This is because the control condition was introduced to ascertain that any issues or inaccuracies with the full model were due to the nature of the recognition algorithm rather than to problems with the virtual agents. Hence, to eliminate any doubts about the suitability of a virtual agent-only dataset to train a gesture recognition algorithm from the start, the evaluation metrics for the control model are presented in Table 5.

**Table 5:** Testing results according to configuration of the control model after training on the control dataset

Configuration	Accuracy	Precision	Recall	F1
**MediaPipe default**	0.783	0.799	0.702	0.797
**MediaPipe example**	0.743	0.917	0.551	0.724
**ChatGPT**	0.858	0.949	0.714	0.849
**Blackbox AI**	0.859	0.948	0.718	0.841

As seen in Table 5, the control model performs differently depending on the configuration. The two hyperparameter value combinations suggested by MediaPipe perform the worst overall, reaching accuracy of only 0.783 and 0.743 for default settings and model example, respectively. Additionally, the recall for the MediaPipe model example configuration only reaches R = 0.551, implying that 44.9% of all true positives remain unrecognised by the model. The two configurations suggested by the AI chatbots performed noticeably better in all metrics except precision (where they performed on par with the MedaPipe example but not with the MediaPipe default). The combination of hyperparameter values recommended by Blackbox AI was most accurate, with categorical accuracy of 0.859, a precision score of P = 0.948, and a recall score of R = 0.718, resulting in an F1-score of F1 = 0.841. ChatGPT’s recommendations for the hyperparameter values resulted in similar results, with categorical accuracy of 0.858 and an even higher F1-score than BlackboxAI of F1 = 0.849, resulting from a precision score of P = 0.949 and a recall score of R = 0.714. It is striking that the precision scores—percentage of predicted positives being true positives—are above 0.9 for three out of four hyperparameter settings. The combination of a high precision score and a much lower recall score indicates that the model is reluctant to predict a handshape class, but when it does predict a class, it is the correct one in over 90% of cases.

As expected, the models trained on the full dataset do not perform as well as the control models. The evaluation results are summarised in Table 6.

**Table 6:** Testing results according to configuration after training on the full dataset

Configuration	Accuracy	Precision	Recall	F1
**MediaPipe default**	0.594	0.869	0.386	0.548
**MediaPipe example**	0.598	0.816	0.302	0.557
**ChatGPT**	0.687	0.931	0.471	0.647
**Blackbox AI**	0.716	0.931	0.512	0.702

As seen in Table 6, the pattern seen in the control models of the MediaPipe configurations performing worse than the AI chatbots’ configurations can also be observed for the full models. Again, MediaPipe’s default settings and model example hyperparameter values perform the least accurately, with a categorical accuracy score of 0.594 and 0.598, respectively. What is especially striking is that only around a third of all true positives were discovered by the models based on the hyperparameter values provided by MediaPipe, as seen by the low recall scores of R = 0.386 for the default MediaPipe and R = 0.302 for the MediaPipe example. Using the configurations recommended by the two chatbots significantly improves the recall scores to R = 0.471 for the configuration recommended by ChatGPT and R = 0.512 for the configuration recommended by Blackbox AI. Despite this improvement, the recall scores still imply that around 50% of the true positives in the dataset are not recognised by the models. However, overall, even in the less accurate models, the precision scores of P = 0.869 for the default model, P = 0.816 for MediaPipe’s example model, and P = 0.931 for both ChatGPT’s configuration and Blackbox AI’s configuration suggest that the majority of predicted positives are true positives for all models. Similar to the control model, the combination of a high precision score and a much lower recall score indicates that the model is reluctant to predict a handshape class, but when it does predict a class, it is the correct one in the majority of cases. Overall, as with the control model, Blackbox AI’s recommendations perform best for the full model, with the same precision score as ChatGPT’s recommendations (P = 0.931) but a higher recall score (R = 0.512 vs ChatGPT’s R = 0.471), resulting in a higher F1-score (R = 0.702 vs ChatGPT’s F1 = 0.647).

#### Discrepancies in accuracy per handshape

After looking at the models’ overall performance, we closely examined the best-performing models by testing their accuracy per handshape category. For the control condition, BlackboxAI’s configuration performed the best, with an overall weighted accuracy score of 0.859. Per class, the model shows a precision, recall, and F1-score of 0.84 for open palm, along with P = 0.84, R = 0.86, and F1 = 0.85 for thumb tucked, as well as P = 0.89, R = 0.91, and F1 = 0.90 for enclosed thumb and P = 0.99, R = 0.64, and F1 = 0.78 for none. This is most likely due to the distractor data being the least represented class in the overall dataset (i.e., 360 distractor frames compared to 2,000–3,000 frames for each target handshape), resulting in the model being fed fewer training examples including distractors. Additionally, the distractor data contained several different handshapes, making it the least homogeneous category in the set. Therefore, the model does not seem to learn the distractors very efficiently, resulting in a worse performance for the ‘none’ class than for the other three handshapes classes. This explanation is further supported by the same issue, albeit not as strikingly, being observed for the full model. The model trained on the full dataset has an overall weighted accuracy score of 0.716. Per class, the model shows a P = 0.71, R = 0.79, and F1 = 0.75 for open palm, along with P = 0.63, R = 0.78, and F1 = 0.70 for thumb tucked, P = 0.92, R = 0.56, and F1 = 0.70 for enclosed thumb, and P = 0.85, R = 0.54, and F1 = 0.66 for none. Overall, both the control model and the full model perform the worst on the distractor handshapes. However, there is no discernible pattern for the remaining handshape classes, and further research is needed to explain any discrepancies between classes (see Table 7).

**Table 7:** Per-handshape test results for best-performing model (BlackBoxAI)

Handshape	Precision	Recall	F1	Precision	Recall	F1
	Control model			Full model		
**None**	0.99	0.64	0.78	0.85	0.54	0.66
**Open palm**	0.84	0.84	0.84	0.71	0.79	0.75
**Thumb tucked**	0.84	0.86	0.85	0.63	0.78	0.70
**Enclosed thumb**	0.89	0.91	0.90	0.92	0.56	0.70

#### Application

To test whether the virtual agent-trained model generalises to human data, we created a new test dataset, using photographs of the authors producing the same handshapes. This dataset consisted of 432 images: 4 handshapes × 3 people × 2 hand orientations (vertical or sideways) × 3 hand positions (overhead, head-level, shoulder-level) × 3 camera angles (centred, 45* left, 45* right) × 2 (i.e., each of these sets was created twice, at separate times and with different backgrounds, and varying the use of left and right hand, to include more variation). We provided these images to the trained models (BlackboxAI full model and BlackboxAI control model) and used the predicted handshape classes to again calculate accuracy, precision, recall, and F1 scores for each handshape. Table 8 provides an overview of the per-handshape results. Per-handshape results are given as these are most relevant as a test case of this methodology. This is because the accuracy for the distractor (i.e., ‘none’) class is less relevant in general, and there are far fewer distractor items, with much greater variability in the distractor handshapes.

**Table 8:** Per-handshape test results for human image test

Handshape	Accuracy	Precision	Recall	F1	Accuracy	Precision	Recall	F1
		Control model				Full model		
**None**	.02	0.67	0.02	0.04	0.03	0.67	0.02	0.04
**Open palm**	.77	0.77	0.67	0.71	0.91	0.78	0.69	0.73
**Thumb tucked**	.68	0.66	0.55	0.60	0.72	0.84	0.53	0.65
**Enclosed thumb**	.92	0.49	0.78	0.60	0.95	0.54	0.69	0.61

Beyond per-handshape testing, we also tested the model on an open SfH dataset (Shafique, 2023) containing still frames from video recordings. The dataset, the only openly available SfH dataset at the time of writing, contains 28 videos (1,761 frames) without the SfH gesture and 36 videos (5,362 frames) containing the SfH gesture. The recordings vary from only having an arm in frame with a neutral background, to showing a full individual or showing multiple individuals in a public space, with the SfH and/or other hand gestures appearing at different times. It also utilises a variety of lighting qualities and subjects. For this application test, we assessed whether, for each video, a sequence of thumb tucked to enclosed thumb is detected. This two-handshape sequence is used because, contrary to most SfH displays that start with an open hand where the thumb is slightly extended to be next to the palm and index finger (see Fig. 1), this dataset typically started with an ‘open palm’ handshape where the thumb was slightly tucked into the palm, but not fully bent laterally across the palm. Despite the difference in this dataset from typical SfH displays, and our own training data, we include this as an extra test of our models due to the diversity in hand presentation. This test provides a measure of not only frame-wise accuracy, but also the stability of detection, as it requires the model to detect an uninterrupted sequence of the handshapes. The BlackboxAI full model performed with accuracy of 0.73, precision of 1.0, recall of 0.53, and F1 of 0.69. The ChatGPT control model performed with accuracy of 0.61, precision of 1.0, recall of 0.31, and F1 of 0.47. The results of the full model are highly similar to the validation results presented in Table 6, as well as the human data testing from Table 8, showing strong generalisability. Notably, the results of the model trained on the control dataset are much lower, highlighting the advantage of the diversified dataset when testing on real-world, challenging data.

## Discussion

**Fig. 7:**
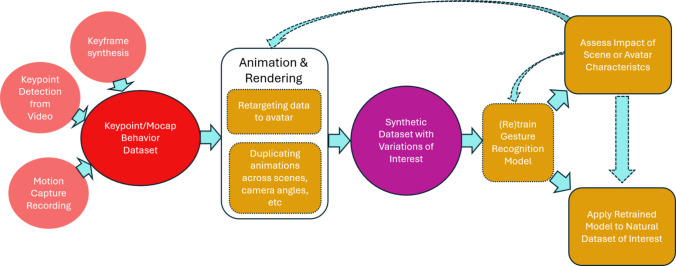
Overview of proposed pipeline. From left to right, we see the creation of a motion tracking dataset, which can be created in several ways. Animation and rendering of videos (using a variety of customisation options) leads to a synthetic gesture dataset. This synthetic dataset can then be used to (re)train a gesture recognition model. Evaluating model performance allows one to (1) assess the impact of particular characteristics that are systematically varied during the animation and rendering stage, or (2) directly apply the custom gesture recognition model to one’s own data. Understanding of the impact of particular features can also lead one to retrain the model again to optimise performance, assess other features, or a priori inform data collection design

The current paper set out to assess whether training a gesture recognition algorithm on artificial data generated using virtual agents is a resourceful, convenient, and effective method for researchers in various domains, including multimodal communication research. We show that a gesture recognition algorithm can be retrained on virtual agents and then applied to human participants. Second, we show that the virtual agent generation process allows researchers to manipulate many aspects of the ‘recording’ environment, allowing unprecedented assessment, testing, and potentially model-refinement of many factors related to context, environment, and participant appearance. See Fig. 7 for an overview of this pipeline.

### Suitability of virtual agent-only dataset

Our results support the notion that virtual agents can be used to successfully train a gesture recognition algorithm. As demonstrated by the high accuracy of up to 85.9% obtained by the control model (when validating the model with virtual agent data), a gesture recognition algorithm can indeed be trained on a virtual agent-only dataset and furthermore achieve close to state-of-the-art recognition accuracy. In terms of similar work with a similar number of recognition classes, our approach approximates the results presented by Ding and Su (2023; accuracy of 90.8% on six classes), Liu and Peng (2009; accuracy of 96.2% on three classes), and Moryossef et al. (2020; accuracy of up to 91.0% on two classes), as the control model reaches up to 85.9% on four classes.

We also tested our trained model on photographs of humans producing the same handshapes, across various camera angles and hand positions. Here, model performance is similar to what is seen in the validation of the full model. While the performance metrics are not state-of-the-art, performance is still adequate, and substantially better than chance. This shows that the model is able to transfer from virtual agent training data to images of real humans. Additionally, the fact that recognition performance for the target handshapes is substantially higher than accuracy for the distractor handshapes provides additional evidence that the model has learned from the synthetic data.

It should be noted that, while our model shows somewhat lower accuracy than that of Liu and Peng (2009), we have used a relatively simple, but accessible, gesture recognition model. This ensures that other behavioural researchers are more likely able to adapt this methodology to their own needs. One could of course opt for more advanced, state-of-the-art models and potentially improve model performance further. Additionally, we used relatively non-lifelike virtual agents, which makes the transfer to human data more challenging. Training a model on more state-of-the-art virtual agents, such as MetaHumans, would likely also improve the accuracy of the model when classifying handshapes in human data. Our primary aim, however, was to provide a proof of concept for the use of virtual agents for testing gesture recognition under different conditions, and for addressing data sparsity problems more generally.

#### Benefits

Using fully customised virtual agents, we compiled a dataset consisting of 134,407 images, which could, in theory, have been expanded indefinitely by incorporating more virtual agents, more backgrounds, more angles, etc. This number would most likely not have been possible to achieve by collecting real-life human data only, as the SfHg, despite going viral on social media, is still a niche phenomenon, which is generally only used in vulnerable situations. In particular in such situations where the data include delicate topics or sensitive information, artificial data can also help mitigate potential issues with privacy and/or participant confidentiality. This advantage of synthetic data is one of the driving factors in ongoing research on the use of synthetic data for ML applications in the medical field (e.g., Chen et al., 2021; Rajotte et al., 2022; Paproki et al., 2024).

Furthermore, the use of virtual agents results in a reduced need to rely on participants. In the current study, mocap data for one participant was used to generate eight virtual participants (virtual agents). Despite using the mocap recordings of only one person to create the whole dataset—as previously condemned by Narang et al. (2017)—we created an extensive and diverse dataset by creating artificial variation manually and customising the virtual agents to ensure gender balance and include a variety of skin tones in the dataset. This approach makes the dataset still more diverse than many other datasets used to train ML algorithms. The resulting virtual agent dataset imitates human data so well that it not only approximates results obtained on humans in previous studies, but also causes the same issues as previously observed for human datasets. This finding is not surprising, as existing literature on the use of synthetic data for computer vision applications in domains other than multimodal communication research has also been successful (Paulin & Ivasic-Kos, 2023). As a strong case for creating diverse synthetic training data, we also found that the model trained on the full dataset performed much better on a more challenging open dataset that included different camera distances, lighting, and social scenes, when compared to the control model.

As stated earlier, researchers can use the ability to customise aspects such as lighting conditions and backgrounds to either avoid issues with recognition algorithms or, alternatively, purposefully create extreme conditions to test the robustness of the same algorithms. In the use case presented in the current study, we explored both options. For the control model, we adjusted the background to be neutral and the lighting conditions to be well lit, therefore avoiding potential issues. For the full model, we used both cluttered backgrounds and non-optimal lighting conditions, therefore testing the robustness of MediaPipe’s pretrained gesture recognition model. As expected from previous findings in the literature (e.g., Shanthakumar et al., 2020; Pouw et al., 2020; Kurakin et al., 2012), the control model recognised gestures significantly more accurately than the full model. Just as numerous researchers have found that difficult lighting conditions and cluttered backgrounds cause the greatest issues with recognition accuracy on human data (e.g., Nyatsanga et al., 2023; Sanchez-Brizuela et al., 2023; Nolte et al., 2023), we found that the same aspects strongly affect the recognition accuracy for algorithms trained on artificial virtual agent data. This is illustrated by the strong decline in accuracy from the best control model to the best full model—from 85.9% accuracy to 71.6% accuracy—and even more so by the recall scores, which declined from 71.8% for the best control model to only 51.2% for the best full model. Conducting such tests is greatly facilitated by a virtual agent approach, as controlling for such environmental factors using only human data would have been far more time-consuming, if at all possible.

### Hyperparameter tuning

The goal of this paper is not to discuss hyperparameter strategies at length. However, our comparison of hyperparameter configurations as provided by MediaPipe documentation and querying commonly used generative chatbots provides some useful insights regarding how one should go about tuning their own model.

For both the control model (i.e., ideal lighting and background) and the full model, the results for the best-performing hyperparameter configurations were those provided by ChatGPT and BlackBox AI. The model performance approximated the state-of-the-art gesture recognition results (e.g., Moryossef et al., 2021; Shanthakumar et al., 2020; Sánchez-Brizuela et al., 2023). This suggests that these generative AI applications can also be utilised to provide an initial configuration that could outperform the default settings. Of course, a full-fledged gesture recognition implementation should include fine-tuning of hyperparameter settings to maximise performance.

### Handshape class discrepancies

As shown in the results section, both the control model and the full model perform the worst on the distractor handshapes. However, further research is needed to find patterns and explanations for the discrepancies in performance for the remaining three classes. Given that the three handshape classes open palm, thumb tucked, and enclosed thumb can be considered balanced in the training data, an unequal distribution in the training data can be excluded as the cause for the discrepancies in the performance. Furthermore, when considering the physical properties of each handshape, one might argue it is easier to make a distinction between open palm and enclosed thumb, as well as between thumb tucked and enclosed thumb, than between open palm and thumb tucked. This is because, for the first two combinations, there are four or more fingers changing their position, whereas only one finger—the thumb—changes its position from open palm to thumb tucked. However, such similarity between open palm and thumb tucked could also cause the two handshapes to be recognised more accurately than enclosed thumb, which is less similar physiologically to the other two. Another relevant factor may be that the enclosed thumb handshape involves occlusion of the thumb, while the other two handshapes do not involve occlusion. Due to the limited scope of the current paper, the causes for the discrepancies between the models’ performance for each handshape class cannot be investigated further. However, future studies should examine these differences in more detail to determine the reasons for the divergence in accuracy.

### Practical Application of SfH Detection

The case study used here was to develop a Signal-for-Help gesture detector that would recognise the SfH gesture in diverse scenarios, as produced by a diversity of individuals. Our initial tests show promise for leveraging synthetic data augmented in a variety of ways in order to improve detection in real-world data. However, it should be noted that our models exhibit very high precision but lower recall. This suggests that, as currently implemented, one can be quite confident that each detection is a true case of the SfH being produced. Yet, there are still a sizable number of real cases being missed. For a real-world deployment of such an SfH detector, where the goal is to increase safety, it may be advantageous to also adjust decision thresholds in order to decrease the number of misses, even if that comes with some additional noise in the form of false alarms. Alternatively, while MediaPipe is lightweight and easy to employ, other architectures may provide better performance metrics that outweigh computational costs when being used on a large scale.

### Limitations

The study presented here illustrates the advantages of the proposed methodology of using virtual agents to train recognition algorithms and offers a step-by-step instruction, facilitating replication of the current study as well as the design and implementation of new studies adopting the same methodology. However, there are certain limitations to this approach. Firstly, although the ReadyPlayerMe virtual agents we used already represent humans well and can be considered human-like, they are clearly distinguishable from actual humans. With the technology available at the time of writing, it is also possible to create virtual agents almost indistinguishable from actual humans. Unreal Engine, for instance, recently developed a new virtual agent creation pipeline to build photorealistic digital humans called *MetaHumans* (Epic Games, 2024). The added detail of depth in the hands of the virtual agents could increase accuracy substantially, as many recognition algorithms, including MediaPipe used here, estimate a depth value (*z*-coordinate) in addition to location values (*x*- and *y*-coordinates) during hand landmark estimation. Therefore, using such photorealistic virtual agents instead of the video-game-character-like virtual agents used in the current study should be explored in future studies, as using more realistic virtual agents could result in better recognition accuracy. However, rendering MetaHumans has much higher hardware requirements than the virtual agents used in the current study. As such powerful processors and advanced graphics cards are expensive and not at every researcher’s disposal, we chose not to use MetaHumans in the current project. Additionally, due to the high resolution of the resulting virtual agents, not only is advanced hardware needed, but other resources, such as more power and time to render the virtual agents, are needed as well. In summary, each researcher needs to determine herself whether such photorealistic virtual agents are needed for her research purposes or whether the virtual agents presented in the current study are sufficient.

A further limitation of the current study is basing the whole virtual agent creation process on mocap recordings and not exploring other options, such as keyframe synthesis. As the main purpose of this study was to provide a proof of concept for the use of virtual agents for retraining gesture recognition algorithms and assessing the impact of visual (environmental, contextual) features on recognition, we chose an approach that provides the most high-fidelity synthesis of human movement.

Additionally, the dataset, although already balanced for all handshapes of the SfHg, could have included more distractor frames to eliminate the discrepancy between frames containing the gesture and frames not containing the gesture, potentially leading to higher recognition accuracy.

## Conclusion

We have argued that virtual agents can be used for training recognition algorithms to detect visual cues in human behaviour, in particular gestures, allowing one to synthetically (re)create a particular study design and/or range of participants. We provided a use-case scenario where this approach was used to train a model to recognise a sequence of gestures from a variety of camera angles and under different background and lighting conditions. We believe this methodology can allow researchers investigating visual aspects of human behaviour, in particular multimodal communication researchers, to mitigate problems of data sparsity, and allow researchers to factor in, or quantitatively assess the impact of, contextual features such as lighting conditions, backgrounds, camera angles, or even participants’ visual characteristics.

## Supplementary Information

Below is the link to the electronic supplementary material.

Supplementary file1 (DOCX 153 KB)

## Data Availability

The datasets generated during the current study are available in the ‘Automatic Recognition of the Signal for Help’ repository on the Open Science Framework (OSF), accessible at 10.17605/OSF.IO/8S2MW.

## Code Availability

The code used to train the gesture recognition model and the script for the Gestural Emergency Call prototype are available in the repository linked above.
