# Supplementary material for: Virtual agents as a scalable tool for diverse, robust gesture recognition
Source: Behav Res Methods. 2026 Jan 16;58(2):41. doi: 10.3758/s13428-025-02914-w (PMC12811268; doi:10.3758/s13428-025-02914-w)
Supplement: Supplementary file 1 — Supplementary file1 (DOCX 153 KB) [file 13428_2025_2914_MOESM1_ESM.docx]

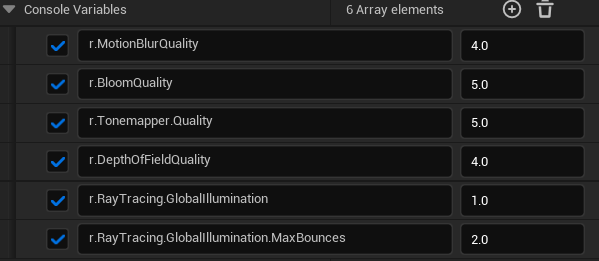


**Supplementary Figure 1**: Additional variables and their corresponding values added to the rendering options in Unreal Engine’s Movie Render

Queue.
